# Multi-Element UWB Probe Optimization for Medical Microwave Imaging

**DOI:** 10.3390/s23010271

**Published:** 2022-12-27

**Authors:** Youness Akazzim, Otman El Mrabet, Jordi Romeu, Luis Jofre-Roca

**Affiliations:** 1 School of Telecommunication Engineering, Universitat Politècnica de Catalunya, 08034 Barcelona, Spain; 2 System of Information and Telecommunications Laboratory (LaSIT), Abdelmalek Essaadi University, Tetouan 93000, Morocco

**Keywords:** microwave imaging systems, brain imaging, medical applications, UWB imaging, focusing technique

## Abstract

The need for non-ionizing techniques for medical imaging applications has led to the use of microwave signals. Several systems have been introduced in recent years based on increasing the number of antennas and frequency bandwidth to obtain high resolution and good accuracy in locating objects. A novel microwave imaging system that reduces the number of required antennas for precise target location appropriate for medical applications is presented. The proposed system consists of four UWB extended gap ridge horn (EGRH) antennas covering the frequency band from 0.5 GHz to 1.5 GHz mounted on a cylindrical phantom that mimics the brain in an orthogonal set of two EGRH probes. This configuration has the ability to control both the longitudinal and transversal dimensions of the reconstructed target’s image, rather than controlling the spatial resolution, by increasing the frequency band that can be easily affected by medium losses. The system is tested numerically and experimentally by the detection of a cylindrical target within a human brain model.

## 1. Introduction

Microwave imaging (MWI) is a technique that employs electromagnetic waves to evaluate the dielectric properties of an object and to detect its location. This has led to its use in medical applications [1,2], as a complementary solution to the use of conventional techniques, such as X-ray mammography, computed tomography (CT scan), ultrasound imaging (US) and magnetic resonance imaging (MRI). MWI has some interesting features, such as the use of non-ionizing energy, low cost, and rapid processing time. The system described in [3] was one of the first MWI systems to be used to detect cerebral edema at a frequency of 2.4 GHz, using a simple head phantom and two applicators to transmit the microwave energy beam through the head model and to compare the received microwave signal with a reference signal. Subsequently, different systems were developed for imaging parts of the human body using a higher number of antennas that can reach up to 64 probes, or using a single rotating antenna around the body part resulting in the collection of substantial amounts of data that can include up to 360 measurements. These systems can be classified into two groups:

1. Multi-static antenna systems, which are based on using a large number of probes to illuminate the body parts under study, such as the system described in [4] that was developed for brain stroke detection based on 32 or 64 transceivers at 1 GHz. A total of 10 or 12 patch antennas are mounted on a helmet [5] enabling differentiation between hemorrhagic and acute stroke patients, as well as hemorrhagic from healthy volunteers. A system has recently been developed based on an array of 24 printed monopole antennas placed conformally to the upper part of the head [6,7]. Other systems have been developed for breast cancer detection based on the contrast between healthy and malignant tissue. In [8], a more comfortable system of 16 flexible antenna embedded in a bra for a frequency band from 2 GHz to 4 GHz was developed. Subsequently, a hand-held impulse radar detector was developed for the detection of breast tumors [9] using 16 antennas for a frequency range of 3.1 GHz to 10.6 GHz. In [10], a mm-wave imaging prototype was presented for early breast cancer detection based on a synthetic array of 24 probes, achieved by translating two antennas. The system was used to locate a neoplastic cylindrical model at different depths inside the phantom up to 3 cm using a frequency band of 18 Ghz to 40 Ghz. Other parts of the human body were also investigated, such as the forearm [11], knee [12], skin [13], etc.
2. Monostatic systems based on a single rotating antenna around the body part that transmits and receives signals to increase the number of scanning angles. This technique has been used for brain imaging [14] to detect intracranial hemorrhage using a single compact UWB antenna with a frequency band of 0.75 GHz to 2.55 GHz. Additionally, traumatic brain injuries were investigated in [15], with a system based on a fixed antenna that illuminates a rotating head model for a frequency band of 1.1 GHz to 3.4 GHz. In [16], the breast was investigated using a system in which the antenna is attached to a robotic arm that rotates and moves in a vertical axis to allow the reconstruction of 3D images for a frequency band from 2.5 GHz to 15 GHz.

In UWB microwave imaging, spatial resolution may be understood as the combination of the resolution along the direction of propagation, known as longitudinal resolution, which results from frequency bandwidth extension—the larger the bandwidth, the smaller (better) is the longitudinal resolution—and the resolution transversal to the direction of propagation, resulting from the angular extension of the front-wave—the larger the number of illuminating antennas, the smaller (better) is the angular extension. In general, the objective is to obtain the two resolutions as close as possible. In this paper, it is shown that a combined optimal spatial resolution can be obtained, while preserving an attainable frequency bandwidth and employing a reduced number of illuminating encircling antennas around the biological target. An optimization of the number of probes for the proposed system is presented, starting with two antennas forming an arc around a cylindrical phantom that mimics the brain and then increasing the number of probes to four. The optimal setup for high resolution is obtained with four antennas distributed in an orthogonal set of two EGRH to simultaneously control the two dimensions of the spatial resolution.

The proposed multi-antenna system is able to precisely locate objects within a phantom using a multi-frequency image combination [17] and a reduced number of antennas located in an optimized set of angular positions. The main advantage of this system is its ability to obtain a high level of accuracy in locating objects in different positions with a minimum number of antennas, reduced size, cost, and data-processing time.

This paper is organized as follows: In Section 2, an analytical study of the optimal antenna distribution of the MWI system is described. Section 3 presents the results of numerical and experimental validation of the optimal set of UWB antennas. Finally, the conclusions are presented in Section 4.

## 2. Optimal Distribution of Antennas for the Proposed Cylindrical MWI System

**Figure 1:**
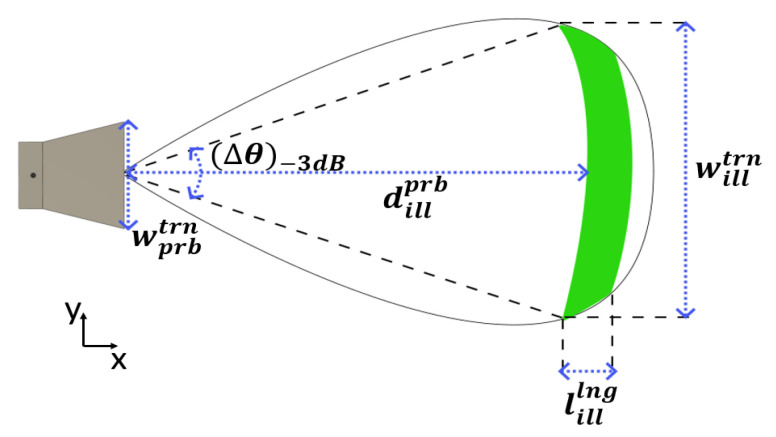
The illumination area of the UWB antenna for microwave imaging.

This section presents the proposed technique for finding the optimal distribution of antennas for the MWI system to be used for medical applications, in particular, investigations of the human brain. An illumination area is illustrated in Figure 1 at a distance of $d_{ill}^{prb}$ from the probe antenna, where $w_{ill}^{trn}$ and $l_{ill}^{lng}$ are the transversal and longitudinal dimensions, respectively. The analytical modeling of the illumination area is presented in (1) and (2). ${\left(\Delta \theta \right)}_{-3dB}$ is the −3 dB beamwidth of the horn antenna, assuming operation in the far field and a moderate non-uniform distribution approached by the ratio between $\lambda_{m}$ and the width of the aperture of the antenna $w_{prb}^{trn}$ [18].

(1) $$w_{ill}^{trn}=d_{ill}^{prb}.{\left(\Delta \theta \right)}_{-3dB}≃d_{ill}^{prb}.\frac{\lambda_{m}}{w_{prb}^{trn}}$$

(2) $$l_{ill}^{lng}=\Delta t.c_{m}=\frac{1}{\Delta f}.c_{m}$$

where $c_{m}$ and $\lambda_{m}$ are the wave velocity and wavelength in the brain medium, respectively.

**Figure 2:**
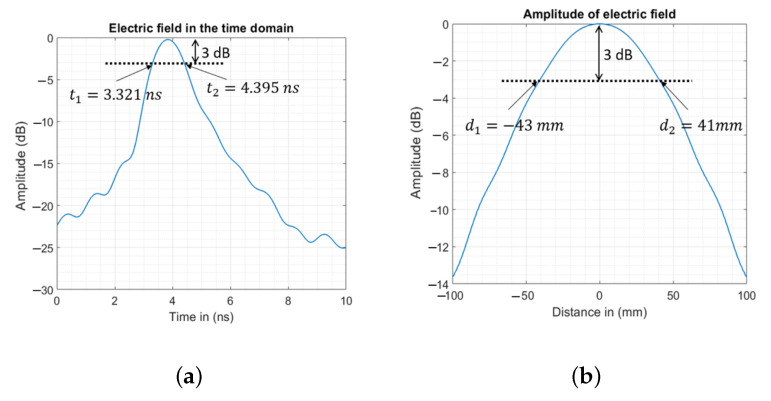
Numerical dimensions of the illuminating area of the UWB antenna used. (**a**) The electric field received at $d_{ill}^{prb}$ from the probe in the time domain. (**b**) The width of the propagated wave at −3 dB from the maximum amplitude at a distance of $d_{ill}^{prb}$.

The dimensions of the illumination area are calculated first with (1) and (2), and then numerically validated using a single UWB antenna with a frequency bandwidth of 0.5 to 1.5 GHz, radiating in a cylindrical phantom that mimics the medium of the human brain. The longitudinal dimension $l_{ill}^{lng}$ corresponds to the product of the wave velocity in the brain medium and the time pulse generated by the UWB antenna, which corresponds to the electric field received at $d_{ill}^{prb}=100.0$ mm from the probe (see Figure 2a) in the time domain. The transversal dimension $w_{ill}^{trn}$ is the width of the propagated front wave at −3 dB from the maximum amplitude at a distance of $d_{ill}^{prb}$ (see Figure 2b).

The analytical and numerical results for these dimensions are presented in Table 1.

**Figure 3:**
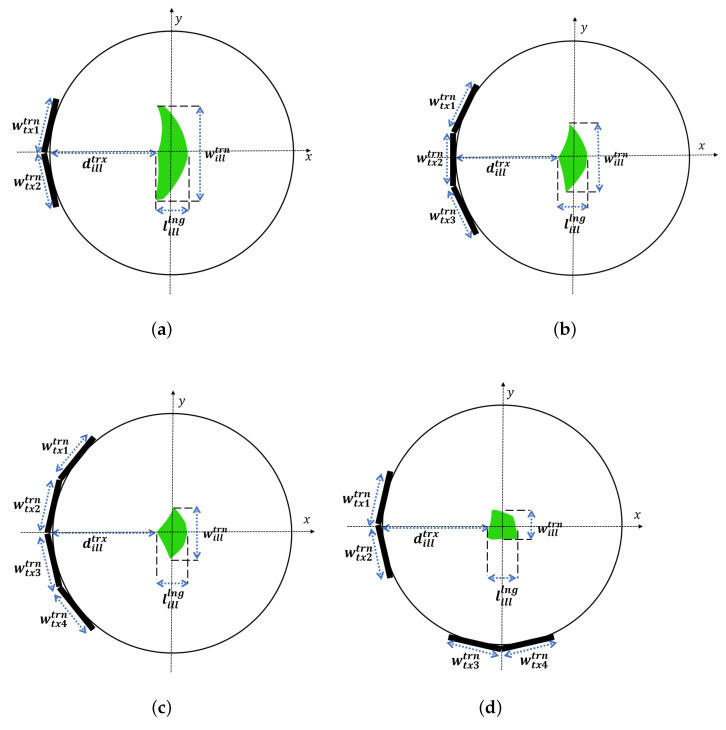
Geometric analysis of image resolution. (**a**) Two UWB antennas. (**b**) Three UWB antennas. (**c**) Four UWB antennas. (**d**) The orthogonal set of two antennas.

To obtain an optimal distribution of antennas for the MWI system, four setups were initially proposed, as shown in Figure 3. Based on the dimension results of the illumination area in Table 1, a geometrical analysis of all the setups was carried out. When using two antennas (Figure 3a), the intersection of the illumination areas generated by the probes creates a wide spatial resolution. The increase in the number of antennas improves the transversal resolution, as shown in Figure 3b,c, which led to the proposed orthogonal set of two antennas (see Figure 3d) to improve the longitudinal dimension, rather than increasing the frequency band that is easily affected by losses in the medium.

**Figure 4:**
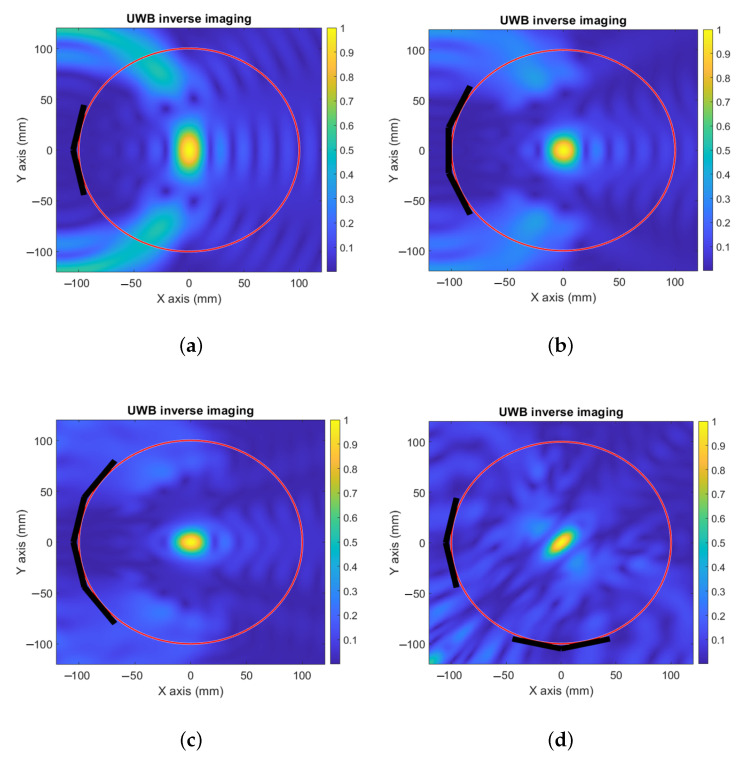
Analytical validation of the inverse imaging. (**a**) Two UWB antennas. (**b**) Three UWB antennas. (**c**) Four UWB antennas. (**d**) The orthogonal set of two antennas.

The analyses presented in Figure 3 are numerically validated using Matlab to calculate the field scattered by an electrically thin metallic cylindrical target at a distance of $d_{ill}^{prb}$ from the probes. The results are then processed to reconstruct the image for the four setups. The best resolution for the transversal and longitudinal dimensions is obtained with the orthogonal set of two antennas (see Figure 4d), which shows good agreement with the geometric analysis in Figure 3.

## 3. Numerical and Experimental Optimization of the Proposed System

**Figure 5:**
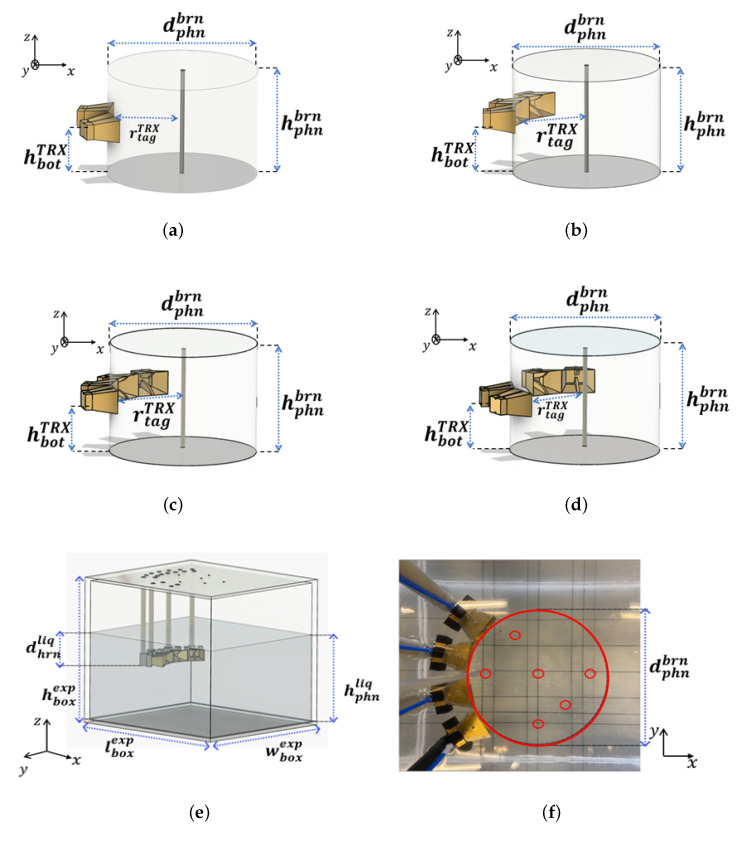
Simulated and measured setups. (**a**) Simulated system based on two UWB antennas. (**b**) Simulated system based on three UWB antennas. (**c**) Simulated system based on four UWB antennas. (**d**) Simulated system based on an orthogonal set of two UWB antennas. (**e**) 3D illustration of the measured setup. (**f**) Top view of the measurement setup.

In this section, an optimization of the systems proposed in the previous section for a precise target location is performed, first by simulations using CST Microwave Studio, and then validated experimentally. The different tested configurations are presented in Figure 5. The study reported in [19] provides accurate head dimensions based on analysis of various human heads, where the average shape is reported to be an ellipse with an overall size of 195.0 mm × 145.0 mm and a height of 142.0 mm, leading to an approximate head model represented by a cylinder of diameter $d_{phn}^{brn}=200.0$ mm and height $h_{phn}^{brn}=150.0$ mm to approximate the real size of the human head. This is illuminated by the EGRH antennas [20] at a mid-height of $h_{bot}^{TRX}=50.0$ mm from the bottom, filled with a liquid material similar to the brain medium with an average permittivity of $\varepsilon_{r}≃57.0$ and σ≃0.6 S/m for a frequency band of [0.5–1.5] GHz [21].

The liquid used for the phantom is obtained by mixing 50.0% of distilled water and 50.0% of methyl alcohol 99.9°. Its complex permittivity was measured using an N1501A dielectric probe kit. A cylindrical metallic target of diameter 3.0 mm is placed within the model at the following (x,y) locations: (−80.0 mm, 0.0 mm), (0.0 mm, 0.0 mm), (80.0 mm, 0.0 mm), (−20.0 mm, 50.0 mm), (0.0 mm, −80.0 mm), and (60.0 mm, −50.0 mm). In the event of detecting a dielectric target with the same system sensitivity and dimensions, the minimum measurable contrast would be of the order of 2%.

The simulated setups using two, three, or four antennas, and the orthogonal set of two UWB antennas are presented in Figure 5a–d, respectively.

In Figure 5e an illustration is presented of the experimental validation of the optimal probe distribution. The EGRH antennas are held on a cover of a methacrylate box of dimensions $l_{box}^{exp}=450.0$ mm, $w_{box}^{exp}=400.0$ mm, and $h_{box}^{exp}=400.0$ mm oriented to the center of a virtual cylinder of diameter $d_{phn}^{brn}$, as shown in Figure 5f. The probes are immersed in a liquid phantom with a depth of $d_{hrn}^{liq}=50.0$ mm, to ensure that the distance from the probe to the liquid upper level is larger than $\frac{\lambda_{eff}}{2}$ for the lowest frequency, where $\lambda_{eff}$ is the effective wavelength. The box cover contains holes for the cylindrical antenna supports to form the different imaging setups proposed in this paper and other holes to insert the target.

To perform these measurements, the probes are connected to a 4-port R&S ZNA vector network analyzer to measure the differential S parameters for both cases, without the target and with the target inserted at the six positions, being the system previously calibrated at the output of each probe antenna.

**Figure 6:**
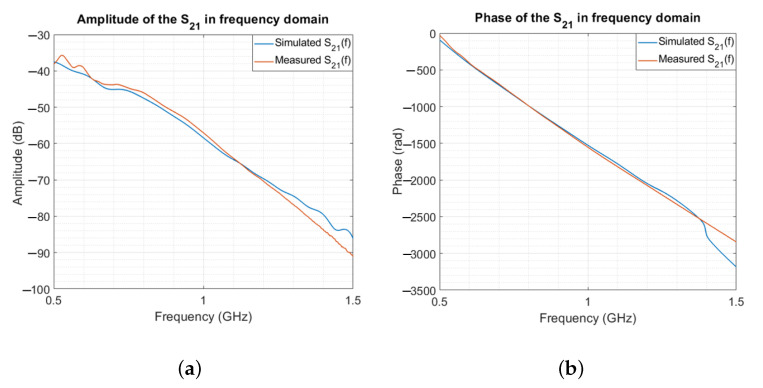
Simulated and measured $S_{21}$ in the frequency domain. (**a**) Amplitude. (**b**) Phase.

In Figure 6, the simulated and measured $S_{21}$ are compared in terms of amplitude (Figure 6a) and phase (Figure 6b). The numerical and experimental results are in agreement throughout the frequency band of interest. The average percentage difference for the amplitude and phase is around 2.5%.

**Figure 7:**
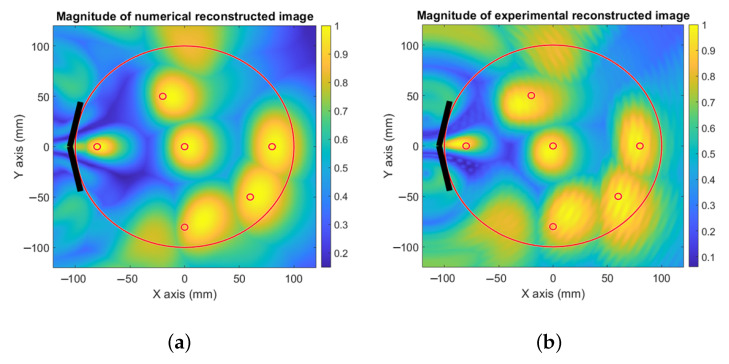

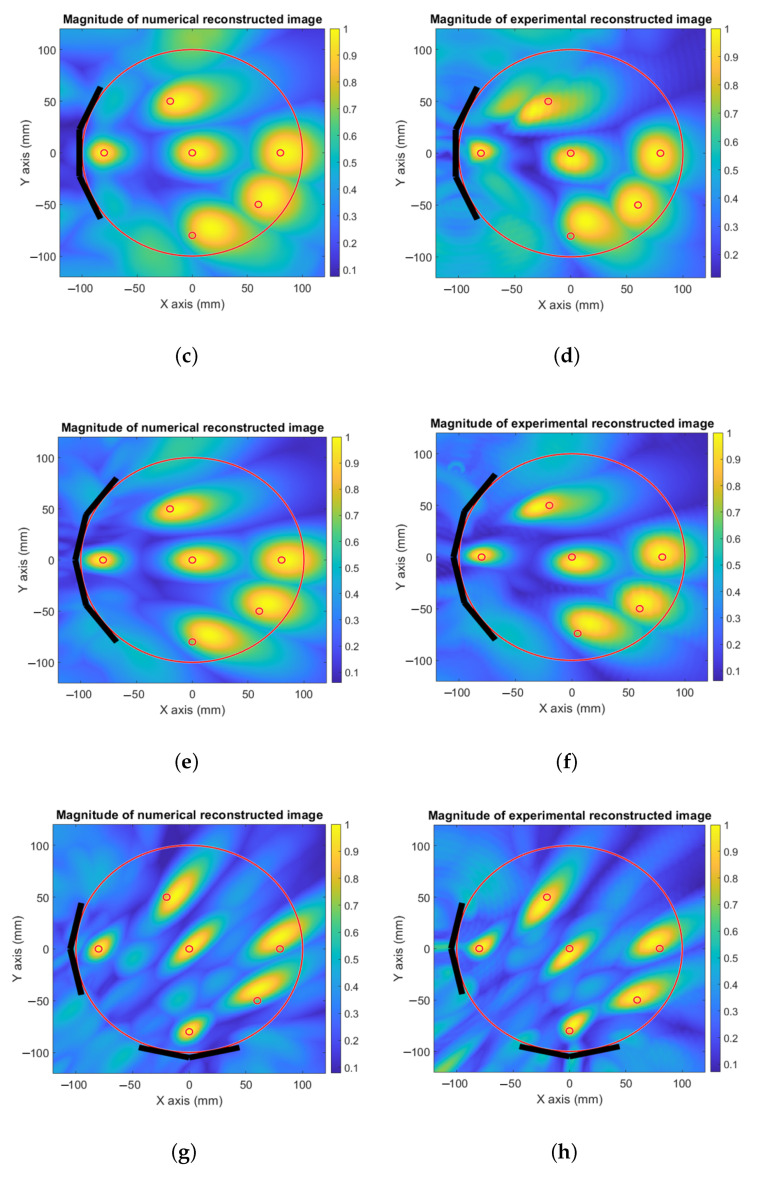
Numerical and experimental image reconstruction of optimized setups. (**a**) Simulated image using a system based on two probes. (**b**) Measured image using a system based on two probes. (**c**) Simulated image using a system based on three probes. (**d**) Measured image using a system based on three probes. (**e**) Simulated image using a system based on four probes. (**f**) Measured image using a system based on four probes. (**g**) Simulated image using a system based on the orthogonal set of two UWB antennas. (**h**) Measured image using a system based on the orthogonal set of two UWB antennas.

The reconstructed images from the numerical and experimental validation are presented in Figure 7, where the received signals produced from different target locations are individually processed and normalized. The images reconstructed using the multi-frequency bi-focusing (MFBF) algorithm presented in [17] are then summed in a single image. For the first setup that contains two EGRH probes as transceivers, the results for simulated (Figure 7a) and measured (Figure 7b) images are able to locate the target precisely within the head model, even though the spatial resolution is broad. In Figure 7c,d, an additional antenna is used that reduces the transversal resolution of the system, as explained in the previous section. When using four EGRH probes that form an arc around the phantom (see Figure 7e,f), the transversal resolution is reduced, but the longitudinal resolution is not affected, as long as the frequency band used is not changed.

To control both dimensions of the spatial resolution, we used an orthogonal set of two UWB antennas (see Figure 7g,h). The results were sufficiently good to precisely locate the objects within the phantom zone, for both numerical and experimental validation, with high resolution for the given frequency bandwidth. The resolution that can be achieved is around $0.6\lambda_{eff}$ in the transversal direction and around $0.7\lambda_{eff}$ in the longitudinal direction.

## 4. Conclusions

A novel optimized multi-element system based on a UWB probe is presented for a microwave imaging system composed of four EGRH antennas forming an orthogonal set of two EGRH probes. This configuration enables simultaneous control of the transversal and longitudinal dimensions without increasing the frequency band that can be easily affected by losses presented in the medium. The numerical and experimental validation conducted shows good agreement with the proposed analytical approach for the optimal distribution of antennas. This system is appropriate for medical applications based on the precise detection of targets in human body parts, such as tumor detection or location of the origin of brain signals for brain monitoring, using a minimum number of antennas to reduce the size, cost, and processing time.

## Figures and Tables

**Table 1:** Analytical and numerical dimensions of the illumination area.

	$w_{\mathrm{ill}}^{\mathrm{trn}}$ in mm	$l_{\mathrm{ill}}^{\mathrm{trn}}$ in mm
Analytical	88.3	39.7
Numerical	84.0	42.7

## Data Availability

Not applicable.
